# Single-Cell Technologies for the Study of Antibody-Secreting Cells

**DOI:** 10.3389/fimmu.2021.821729

**Published:** 2022-01-31

**Authors:** Matteo Broketa, Pierre Bruhns

**Affiliations:** ^1^ Institut Pasteur, Université de Paris, INSERM UMR 1222, Unit of Antibodies in Therapy and Pathology, Paris, France; ^2^ Sorbonne Université, Collège doctoral, Paris, France

**Keywords:** plasma cell (PC), high-throuput technique, antibody secreting cell, droplet microfluidics, antibodies, B cells, functional bioassay

## Abstract

Antibody-secreting cells (ASC), plasmablasts and plasma cells, are terminally differentiated B cells responsible for large-scale production and secretion of antibodies. ASC are derived from activated B cells, which may differentiate extrafollicularly or form germinal center (GC) reactions within secondary lymphoid organs. ASC therefore consist of short-lived, poorly matured plasmablasts that generally secrete lower-affinity antibodies, or long-lived, highly matured plasma cells that generally secrete higher-affinity antibodies. The ASC population is responsible for producing an immediate humoral B cell response, the polyclonal antibody repertoire, as well as in parallel building effective humoral memory and immunity, or potentially driving pathology in the case of autoimmunity. ASC are phenotypically and transcriptionally distinct from other B cells and further distinguishable by morphology, varied lifespans, and anatomical localization. Single cell analyses are required to interrogate the functional and transcriptional diversity of ASC and their secreted antibody repertoire and understand the contribution of individual ASC responses to the polyclonal humoral response. Here we summarize the current and emerging functional and molecular techniques for high-throughput characterization of ASC with single cell resolution, including flow and mass cytometry, spot-based and microfluidic-based assays, focusing on functional approaches of the secreted antibodies: specificity, affinity, and secretion rate.

## Introduction

Antibody-secreting cells (ASC) are B cells that have differentiated following activation to secrete various soluble isotypes of their immunoglobulin receptor with the purpose of binding their target antigen throughout the body (1). ASC are predominantly generated within the germinal center reactions of secondary lymphoid organs (2), although extra-follicular responses may also generate ASC (3, 4). Following antigen exposure, parallel downregulation of major regulatory genes of activated B cells and upregulation of a unique ASC transcriptional program drives differentiation of B cells into early-ASC or plasmablasts. Several mechanisms have been proposed to govern ASC fate determination, but a unifying model has not yet been determined (1, 5). Plasmablasts are an unstable ASC intermediate that require input from a survival niche to persist long term. The bone marrow has been extensively studied as an ASC niche for its role in harboring ASC following infection and immunization, however the majority of ASC are located in the gut-associated lymphoid tissue and produce IgA (6); the thymus has also recently emerged as an ASC niche (7, 8). The factors involved in early-ASC homing to survival niches are not completely understood. Plasmablasts may also be drawn towards sites of inflammation, where they act locally and acutely without persistence (9). Plasma cells are a long-lived ASC subset, characterized by reinforced expression of genes within the ASC differentiation network, responsible for secreting large quantities of antibodies from within their survival niches.

**Figure 1:**
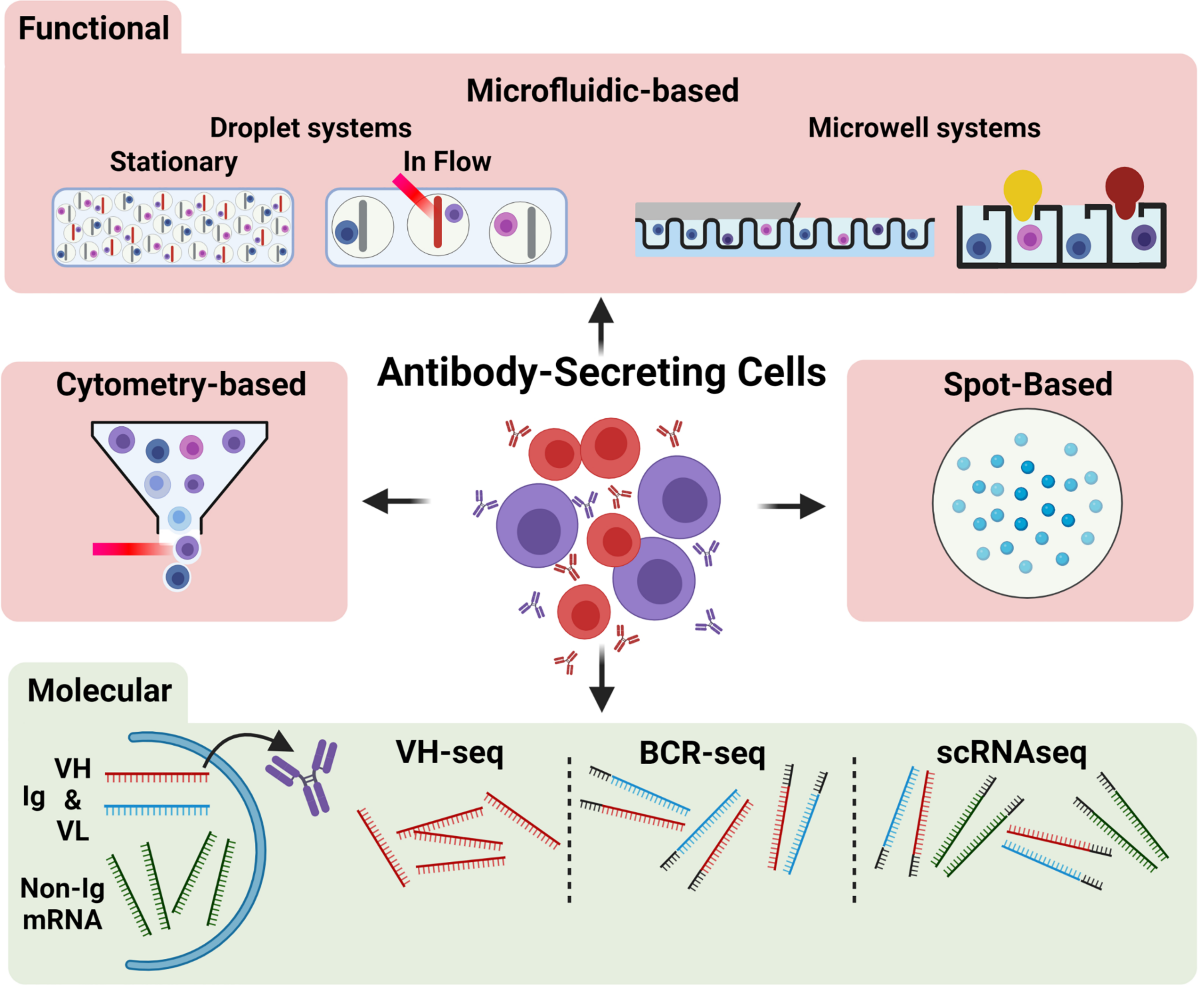
Overview of common techniques for single ASC characterization. Antibody secreting cells (ASC) may be characterized functionally (red region) or molecularly (green region). Functional methods include microfluidic approaches (top), including stationary and flowed droplet-based systems and microwell systems (far right: Berkeley Lights Beacon setup), cytometry-based approaches, and spot-based assays. The red streak represents a laser beam; the yellow and dark red bulb shapes indicate a positive microwell). Molecular methods (bottom) may assess the V_H_ (red band) and V_L_ (blue band) antibody-encoding mRNA transcripts or non-antibody related mRNA transcripts (green bands). Molecular approaches commonly amplify VH chains only (e.g., VH-seq), V_H_ and V_L_ chains with the addition of barcodes (grey bands) or linkage (e.g., BCR-seq), or all mRNA within the cell (e.g., scRNAseq).

ASC are often considered the “apex” of B cell differentiation as they actualize antibody-mediated humoral immunity and are terminally differentiated. ASC contribute to both the acute humoral response to infection by rapidly generating early antibodies at sites of infection as well as later secreting higher affinity antibodies produced by germinal center reactions to aid in pathogen clearance and protective immunity. While the ASC response is advantageous during infection and when co-opted for immunization, emergence of ASC secreting antibodies towards self-antigens is a deleterious factor in many autoimmune disorders (10). Despite their importance, much is still unknown regarding ASC differentiation, selection, and heterogeneity, particularly in autoimmune disorders. High throughput (HT) analyses of single cells are becoming more accessible, affordable, and common in literature concerning adaptive immune responses. This review will outline current and emerging HT techniques to characterize single ASC (**Figure 1**), with discussion of recent applications of these techniques to study the role of ASC in various pathologies as well as to expand understanding of fundamental ASC biology.

## Functional Analyses

The primary effector functions of ASC are mediated by their secreted antibodies, and thereby are characterized by a secreted phenotype. Polyclonal antibody responses of ASC are routinely assessed by sampling the serum or ASC-containing organs for binding antibodies and their global potency (11). Such methods provide an overview of the cumulative ASC response but are unable to interrogate ASC diversity. Techniques with single cell resolution are required to uncover the contributions of individual clones to the polyclonal ASC pool, the prevalence of specific groups of ASC, and the relationships between the factors and markers that differentiate ASC. It should be noted that many other soluble effectors besides antibodies are also known to be secreted from ASC (12, 13), including various interleukins and transforming growth factor-beta one (TGF-β1), and HT functional assays may also be readily adapted to study the role of these soluble factors.

The topic of techniques for measuring single-cell protein secretion in immunology and their origins have been previously described (14, 15). Three general strategies are available for characterizing the secreted effectors of immune cells, each with applicability to ASC: spot-based assays, cytometry-based assays, and microfluidic-assays. These approaches globally share the strategy of isolating ASC, either by distance or compartmentalization, to ensure that “positive” loci or compartments reflect secretions or markers of single cells. Here we will focus on what these techniques offer to describe ASC behavior and highlight notable demonstrations of their use.

### Spot-Based Assays

Spot-based assays spatially distribute ASC by dilution and rely on localized membrane capture of secreted antibody or cytokine from ASC, followed by visualization with enzyme- or fluorophore-linked secondary antibodies (16, 17), termed enzyme-linked immunospot (ELISpot) and Fluorospot, respectively. The number of “spots” formed is indicative of the prevalence of ASC and/or antigen-specific ASC from a given sample. The strength of spot-based assays are their ease of use, robustness, and versatility between models (18, 19). Spot-based assays also directly observe secretion and therefore robustly identify ASC, in contrast to indirect-functional or molecular techniques where the ASC definition is inferred. However, ELISpot and Fluorospot are limited to the detection of only 1-2 or 1-4 soluble analytes, respectively, cannot precisely quantify secretion rates, and the ASC identified cannot be recovered for further analysis. ELISpot has seen extensive use in characterizing the ASC response during pathogenic infections (19, 20), in autoimmune disorders (21–23), and following immunization (24). Bonezi et al (25) recently used ELISpot to demonstrate the contribution of altered tryptophan metabolism to the hyper-abundance of ASC frequently seen during flavivirid infections, Dengue virus being the most common example.

### Cytometry-Based Assays

Flow cytometry and its derivative, fluorescence-activated cell sorting (FACS), have become a staple in investigations of B cells and ASC, allowing for relatively straightforward and rapid evaluation of multiple samples or experimental conditions (26, 27). Multicolor flow cytometry allows for immense diversity and depth of information gathered through the customization of antibody panels to target surface and intracellular markers. In contrast to ELISpot, flow cytometry can precisely identify the proportions of different ASC, B cell, and lymphocyte populations in each sample. Flow cytometry and ELISpot are frequently used in concert to measure global changes in the proportion of ASC and other B cells and the robustness and antigen-specificity of the ASC response, respectively (28, 29).

Human ASC are often defined in flow cytometry as larger cells with CD27 and CD38^high^ expression among CD3^-^ CD20^-^ cells, and may be subdivided by expression of CD138, CD19, CD45, CD81, HLA-DR, and immunoglobulin isotypes (30–33). Plasma cells secreting human IgA or IgM, but not IgG, express surface immunoglobulin and may be identified by direct surface staining (34, 35). Local capture of IgG antibodies secreted by ASC onto their surface has been proposed to identify human IgG-secreting ASC by flow cytometry, using CD45 as a membrane anchor onto which IgG antibodies are captured by their constant region (Fc) (36), but has not yet been widely used. Surface markers and soluble effectors, namely antibodies and cytokines, not expressed at the cell membrane may be assessed using cell permeabilization and fixation to allow labelling reagents access within the cell; such intracellular staining normally precludes downstream assessment of cells by other functional or molecular methods. However, Price et al (37) demonstrated the feasibility to molecularly characterize ASC following intracellular staining for both antibody isotype as well as antibody specificity using tetramer constructs of antigens bound to fluorophore-conjugated streptavidin. Alternatively, early IgG-expressing ASC (IgG-ASC) often continue to bear their antibodies at the cell membrane, and surface staining for antibody isotype and antigen-specificity with tetramers offers a simpler method for isolating such IgG-ASC, though represents a restricted view of the whole IgG-ASC population (38, 39). Flow cytometry is also a valuable tool for scouting potential novel markers of ASC subsets (40). The use of intracellular tracing dyes further allows for assessment of ASC proliferation, as Scharer et al (41) have applied to ASC emergence from activated B cells.

Flow cytometry has identified ASC and ASC subsets in the context of autoimmune disorders and their contributions to acute and chronic disease states (42–45), and the ASC response to natural infection, as recently shown by Woodruff et al (46) in the context of COVID-19. The authors showed by flow cytometry a predominance of extrafollicularly-activated ASC in circulation, based on varied expression of CD11c, CXCR5, and CD21, and demonstrate its effect on infection morbidity. Noticeably, distinction between ASC derived from germinal centers or extrafollicularly is not possible with most other functional assays and is often overlooked. Application of FACS to *in vitro* models of ASC differentiation (47) and survival (48, 49) also highlights the ability of flow cytometry to distinguish ASC subpopulations. FACS isolation of populations of interest may be followed by a spot-based assay to query the subsequent prevalence of ASC within those populations (50–52).

It should be noted that current cytometric-gating strategies for ASC populations have not been demonstrated to completely define all ASC without exclusion of rarer phenotypes. Strategies often vary considerably, utilize a unique marker that may not be ASC-identifying in all contexts, or rely on intracellular staining of transcription factors (31, 53). ASC are distinct in their transcriptional regulation compared to other B cells, and the transcription factors IRF4, BLIMP-1, and XBP1 are ideal for complete identification of ASC (1, 53). Indeed, BLIMP1-YFP mice have been invaluable to studying ASC differentiation, behavior, and transcriptional regulation (54, 55). IRF4-based labelling has similarly been used to identify differentiating human plasma cells following influenza vaccination (56).

Mass cytometry is based on a similar principle as flow cytometry, using heavy metal ions instead of fluorophores to distinguish the antibodies in the cytometry panel. Glass et al (57) utilized multiple mass cytometry panels to characterize over 350 B cell surface markers, combined with analyses of isotype usage, BCR sequence, metabolic profile, biosynthesis activity, and signaling response, which together constitute an expansive single-cell atlas of human B cells. An additional mass cytometry panel targeting enzymes associated with different metabolic pathways (58) found plasma cells to be highly metabolically active, and further subdivisible based on transcriptional activity; such metabolic distinctions have largely been restricted to molecular studies or classical metabolomics (49, 59). Although mass cytometry can simultaneously detect ~40 parameters per panel, the vaporization of analyzed cells prevents further study of the precise subsets identified, which remains the advantage of FACS.

### Microfluidics

Microfluidic single cell assays essentially miniaturize existing techniques to assess ASC secretions, to increase the scale and throughput of analyses and enable single cell resolution *via* compartmentalization into either wells or droplets (14, 15). Miniaturization also allows for greatly reduced reagent usage and less cells are needed per experiment. The functional measurements within microfluidic ASC assays largely share the concept of capturing secreted antibody or cytokine onto a physical surface followed by visualization with fluorescent detection reagents. The advantages of microfluidic-based functional analyses of ASC include their direct identification of ASC *via* antibody secretion, a high throughput, and the ability to absolutely quantify ASC secretions over time.

Micro- and nano-well microfluidic approaches use microfabrication techniques to create thousands of wells into which individual ASC can be introduced (60–62). The nature of the wells and coating and reagent strategy used can be readily customized to assay different antibodies, antigens, or secretions. The small volumes of these wells allow for absolute quantification of secreted molecules, in contrast to ELISpot where only relative measurements can be made (14). Well-based microfluidic assays also have lower detection thresholds than ELISpot, making them more sensitive and more capable of wholly representing ASC (63). Importantly, as ASC are identified and sustained within the static well array, cells can be recovered following the assay for additional functional or molecular assessment. The Berkeley Lights “nanopen” platform introduces considerable advancement to well-based ASC assays using optofluidics, offering integrated workflows for antibody screening of ASC followed by targeted recovery of specific cells for molecular analysis (64). This rapid pipeline for functional antibody assessment was recently used to characterize antibody repertoires following SARS-COV2 infection (65). Despite their advantages over ELISpot, well-based microfluidic assays have seen limited application beyond technical demonstrations and antibody discovery (66–68), mainly due to their cost and limited throughput.

Droplet microfluidics achieves single cell compartmentalization using two immiscible fluids, an aqueous phase containing the cell and assay reagents and an oil phase that separates the aqueous phase into droplets with single cells (14). Large numbers of droplets can be quickly produced for each new sample and the assay composition can be easily changed. Secretion by encapsulated ASC is assessed by beads or other cells within the droplet that act as capture surfaces for secreted cytokine or antibody, to which fluorescent reagents in the droplet can localize for measurement. Flow-based or flowed droplet assays can characterize ASC with high throughput by passing droplets at high frequency through a laser for rapid detection of fluorescence relocalization within droplets (69–71). Droplet measurement may also be paired with dielectrophoretic sorting, which pushes or pulls droplets into separate channels by manipulating electric fields, allowing for further assessment of directly identified ASC or antigen-specific ASC (69, 72). We reported in Gerard et al (69) the CelliGo assay using a double fluorescent sandwich ELISA in microfluidic droplets for the identification, sorting, and V_H_-V_L_ sequencing of antigen-specific IgG antibodies produced by ASC from immunized mice. In this study, we demonstrated screening of a bacterial antigen (tetanus toxoid), an autoantigen linked to Rheumatoid Arthritis (Glucose-6-phosphate Isomerase), and an insoluble, membrane-expressed antigen (tetraspanin-8; TSPAN8); antigen-specific ASC against all 3 model antigens were able to be sorted by this flowed droplet microfluidic technique.

**Figure 2:**
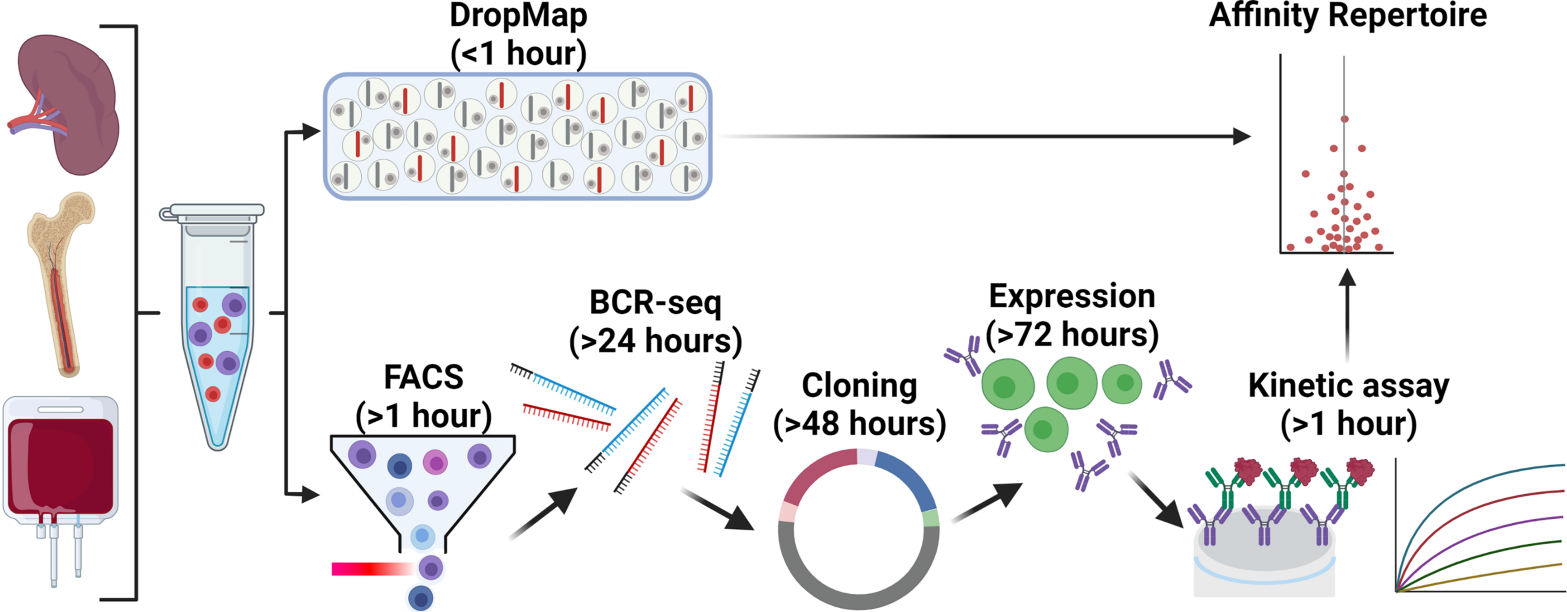
Comparison of DropMap vs a classical pipeline to define affinity repertoires. The affinity repertoire of ASC towards a given antigen is an important metric for the quality of the ASC response. ASC are commonly isolated from the spleen, bone marrow, or blood of donor/patients or experimental animals. The DropMap assay (top) offers a platform that within 1 hour can return the affinity repertoire in a single assay, as well as the IgG secretion rate and frequency of ASC according to direct *ex vivo* measurement (refer to main text for details); this approach is limited by requiring ASC to secrete antibodies at the time of data acquisition, data can only be acquired once, and afterwards cells of interest are lost. An alternative strategy (bottom) to yield a similar affinity repertoire first requires cell isolation by FACS, followed by V_H_ and V_L_ targeted RT-PCR (& BCR-seq if required) and cloning into an expression vector, transfection of the vector into an expression system and cell culture, purification and finally assessment of the recombinant expressed antibody by BLI or SPR; this pipeline requires 1½ -2 weeks to complete, is significantly more complex and costly, and can only assess V_H_-V_L_ pairs that could be successfully amplified, cloned and expressed as recombinant antibodies.

An alternative approach to study ASC in droplets is to collect droplets in a horizontal plane and measure changes in droplet fluorescence over multiple timepoints, termed a stationary droplet-assay (14) or DropMap (73, 74). DropMap measures fluorescence relocation to a central line of antibody-capturing beads aligned using a magnetic field within each droplet. Both secreted antibody relocation and soluble antigen relocation to the beadline are measured. The ability to measure relocation over time within DropMap allows for the determination of both antibody affinity and secretion rate, in addition to determining the proportion of antigen-specific and total ASC within a sample as with other microfluidic techniques. DropMap has been applied to investigate the physiology of ASC in viral infection (75), autoimmunity (74), and fundamental ASC biology (73, 76). In our view, DropMap offers a major advancement in current capabilities to functionally characterize ASC, particularly for defining the specificity and affinity repertoire of ASC. An example alternative strategy to DropMap for affinity repertoire mapping is to sort large numbers of B cells or ASC for their BCR sequences, followed by gene synthesis or direct cloning, re-expression, and kinetic analysis of these antibodies by a technique such as bio-layer interferometry (BLI) (77–79) (**Figure 2**); this approach is significantly more complex, costly, and time consuming than DropMap. Recently however, we reported antigen-specific single cell memory B cell *in vitro* differentiation into ASC that allowed for fast and large scale (~400) affinity measurements by BLI from culture supernatants without costly antibody re-expression (80). Initial sorting for ASC may also bias the antibody repertoire through restrictive FACS gating strategies unless a direct functional assay is used prior to directly select for ASC, whereas DropMap offers an unbiased screen of PBMCs or an enriched B cell pool for ASC. Another novel approach involving BCR sequencing paired with liquid chromatography–tandem mass spectrometry proteomics (81, 82) allows for unbiased molecular assessment of antibodies from the serum, but requires infrastructure and specialist supervision, and is currently largely restricted to the most represented antibodies in the serum.

The major limitation to widespread adoption of functional, droplet-based assays of ASC is their bespoke nature. Although droplet microfluidics is a growing field, with most reagents commercially available, labs must produce their own consumables for droplet production and the apparatus for assay observation, which require specialized equipment and expertise.

## Molecular Analyses

The molecular basis for the behavior of ASC is equally important to understanding ASC physiology. The molecular aspects of ASC most investigated are (i) the heavy and light chain variable regions (V_H_ and V_L_) sequences, formed by gene rearrangements and sequence diversification mechanisms (83), encoding the antigen binding domains of antibodies and (ii) the transcriptional profile of ASCs; these aspects may be probed using bulk V_H_-seq or RNAseq, BCR-seq, and increasingly now single-cell RNAseq (scRNAseq) following either single-cell ASC FACS into 96 or 384 well plates, or microfluidic droplet-based barcoded scRNAseq (e.g., 10xGenomics Chromium). Importantly, BCR sequences can be computationally inferred from scRNA-seq data using open-source libraries (e.g. BraCeR (84), VDJPuzzle (85)), linking antibody sequence (i.e., genotype, clonal information) to transcriptome phenotype. Recent advances on ASC characterization using RNAseq, ATAC-seq and ChIP-seq have been reviewed elsewhere (86), as well as molecular mechanisms leading to plasma cell differentiation from the germinal center reaction (87). Glaros et al (38) highlight the capacity for scRNAseq to identify B cell subsets and observe shifts in differentiation, identifying antigen availability as a key regulator of the plasmablast response.

A common strategy for molecular ASC clonal identification is bulk sequencing of V_H_ regions (37), which allows for large numbers of cells to be assayed without the cost and limitations imposed by a need for single cell approaches. However, the V_L_ sequence information is lost, and it is not possible to re-express the original antibody recombinantly. Alternatively, Price et al (37) recently used bulk RNAseq to identify unique transcriptional profiles for IgG, IgM, and IgA ASC subsets, with the ability to assess both clonality and gene expression. Such approaches offer insight into the clonal diversity of ASC responses but have less resolution than paired VH-VL chain sequencing and have no possibility for functional assessment of clones within the antibody repertoire. When the full V_H_-V_L_ sequence is required, single-cell approaches are required, and currently available techniques for HT BCRseq have been reviewed by Curtis and Lee (88), who highlight advances in V_H_ and V_L_ chain pairing by barcoding (69) or linkage as well as increased cell-throughput through the use microfluidics. Moreover, techniques such as LIBRA-seq (89) or CelliGo (69) allow for assessment of antigen specificity or antigen specificity coupled to antibody secretion, respectively, integrated within their BCRseq pipelines. Wang et al (90) used microfluidics to compartmentalize human plasmablasts as single cells and generate paired V_H_-V_L_ repertoires for direct Fab display on yeast and functional assessment. Notably, Jiang et al (91) utilized a combination of bulk VH-seq, BCRseq, and full scRNAseq to identify autoreactive ASC persisting after treatment with rituximab. Such an approach leverages the complexity and throughput of each technique with the depth of information required to address the authors’ underlying question, saving considerable time, and lowering costs overall. Using the 10x genomics technology, integrating emulsion-based single cell separation with barcoded RNAseq (Chromium), plasmablast-derived mAbs from individuals who received SARS-CoV-2 spike mRNA vaccine were characterized for antigen specificity, epitope mapping and neutralization potential (92).

A final molecular approach worth mentioning is the use of mass spectrometry to assess the relative abundance of specific antibodies in serum or a tissue sample, known as Ab-seq (93). Pairing Ab-seq with any of the RNA-seq approaches above allows for inferences to be made regarding the contribution of specific ASC clones to the polyclonal ASC response, providing invaluable insight into the immunological relevance of ASC clonal diversity. Lee et al (94) utilized Ab-seq to identify potently neutralizing antibodies and ASC clones persisting across multiple exposures to influenza. Ab-seq has also been applied in similar studies for antibodies against norovirus (95) and HIV-1 (96).

## Discussion

### Multiplexing Is the Future for High Throughput Single ASC Analyses

The availability of high throughput methods for the study of ASC at the single cell level has progressed immensely for both functional and molecular characterization. Considerable progress has been made in understanding ASC development and differentiation, but with limited information regarding the relationships between population surface marker expression, transcriptional and metabolic activity, and the functional “quality” of the ASC (affinity, specificity, secretion rate).

The presence of immunoglobulins within all plasmablasts or plasma cells does not exclude the possibility for significant stratification of secretion rates from high to nearly undetectable. As we showed in Eyer et al. (73), antibody secretion by a seemingly homogenous population of ASC may vary by several orders of magnitude. The causes of such secretory diversity remain poorly understood. Current techniques for direct measurement of single cell immunoglobulin secretion (spot- and microfluidic-based assays) are currently unable to distinguish precise B cell populations alone. ASC identified and isolated from direct functional assays have not been assessed afterwards with higher parameter techniques such as flow and mass cytometry or scRNAseq, to interrogate the source of this secretory diversity.

Ultimately, investigations of ASC physiology should strive to employ a combination of the techniques discussed. In the context of antibodies, with only a repertoire of V_H_-V_L_ sequences the contribution and quality of a given antibody to the humoral immune response is difficult to appreciate. Likewise, knowing the affinity or neutralization repertoire of antibodies from ASC without knowing their clonality and molecular basis limits insight into ASC population dynamics and distribution.

Evolution of the existing and emerging methods for HT ASC phenotypic characterization to readily integrate with the various single cell molecular techniques should be an immediate goal to overcome current limitations. HT single cell molecular (scRNA-seq) techniques are becoming more accessible and affordable, with great efforts to increase the fidelity and ease of analysis. HT single cell assays will be central to unravelling how ASC phenotypic markers relate to their developmental stage, antibody affinity, and antibody or cytokine secretion rate, and to what degree these elements are interrelated.

## Author Contributions

All authors listed have made a substantial, direct and intellectual contribution to the work, and approved it for publication.

## Funding

PB acknowledges funding from the French National Research Agency grant ANR-14-CE16-0011 project *DROPmAbs* and ANR-18-CE15-0001 project *Autoimmuni-B*, by the Institut Carnot Pasteur Microbes et Santé (ANR 11 CARN 0017-01), the Institut Pasteur and the Institut National de la Santé et de la Recherche Médicale (INSERM). MB is a recipient of a CIFRE fellowship from the French Association Nationale de la Recherche et de la Technologie (ANRT). None of the sources of funding have an interest in the subject matter or materials discussed in the submitted manuscript.

## Conflict of Interest

The authors declare that the research was conducted in the absence of any commercial or financial relationships that could be construed as a potential conflict of interest. PB is a paid consultant to Regeneron Pharmaceuticals. MB declares that he has no relevant conflicts of interest.

## Publisher’s Note

All claims expressed in this article are solely those of the authors and do not necessarily represent those of their affiliated organizations, or those of the publisher, the editors and the reviewers. Any product that may be evaluated in this article, or claim that may be made by its manufacturer, is not guaranteed or endorsed by the publisher.
